# Supplementary material for: Energy-Related Assessment of a Hemicellulose-First Concept—Debottlenecking of a Hydrothermal Wheat Straw Biorefinery
Source: Molecules. 2025 Jan 29;30(3):602. doi: 10.3390/molecules30030602 (PMC11820640; doi:10.3390/molecules30030602)
Supplement: Supplementary file 1 [file molecules-30-00602-s001.zip › molecules-3354939-supplementary.pdf]

Supplementary material

Table S1. Results and respective standard deviations (STD) of the saturated steam hydrolysis (DM = dry mass; oDM = organic dry mass; WS = wheat straw; n. d. = not determined). Published in 10.1016/j.biortech.2023.130071.

Fraction	DM	oDM	Cellulose	Hemicellulose	Lignin	Acetate	Ash	Protein	Rest[b]
Unit	wt%$_{FM}$	wt%$_{DM}$	wt%$_{DM}$	wt%$_{DM}$	wt%$_{DM}$	wt%$_{DM}$	wt%$_{DM}$	wt%$_{DM}$	wt%$_{DM}$
Raw WS	90.9	92.1	31.3	24.2	18.6	4.5[d]	7.9	2.3	11.1
STD	0.2	0.1	1.3	0.9	0.4	0.2	0.1	0.1	1.7
Washed WS	37.1	94.1	32.2	26.4	20.5	5.0[d]	5.9	2.5	7.7
STD	0.2	0.1	0.5	0.9	0.2	0.1	0.1	0.1	1.1
Hydrolysate	4.5	93.0	5.5	57.9	6.8	7.1	7.0	2.3	13.3
STD	0.1	0.3	0.2	1.2	0.1	0.2	0.3	0.3	1.3
Solubilisation	29.6%	30.3%	3.5%[c]	63.1%	4.8%[c]	70.8%	17.5%	13.5%	24.9%
STD	0.7%	1.0%	0.1%	3.1%	0.1%	1.1%	0.5%	1.5%	3.5%
Recovery[a]	14.5%	14.3%	2.5%	31.8%	4.8%	20.7%	17.3%	13.5%	n. d.
STD	0.3%	0.7%	0.1%	1.1%	0.1%	0.3%	1.1%	2.1%	n. d.
Yield[a]	14.5%	13.5%	0.8%	8.4%	1.0%	1.0%	1.0%	0.3%	n. d.
STD	0.3%	0.6%	0.0%	0.3%	0.0%	0.0%	0.1%	0.1%	n. d.

[a]The yield and the recovery account for the specified components in the hydrolysate DM produced by the experimental procedure; [b]The rest accounts for the unspecified components in the respective DM; [c]Detected in the hydrolysate, not by compositional analysis; [d]Without Soxhlet-extraction.

Table S2: Elemental analysis results. Results and respective standard deviations (STD) for the raw materials and produced hydrolysate dry mass (DM = dry mass; WS = wheat straw). Published in 10.1016/j.biortech.2023.130071.

Element	N	C	H	S	C / N	Protein
Unit	g/kg$_{DM}$	g/kg$_{DM}$	g/kg$_{DM}$	g/kg$_{DM}$		%$_{DM}$
Raw WS	3.7	450.0	60.7	< 2.0	120.6	2.3
STD	0.12	0.00	0.58	0.00	3.80	0.07
Washed WS	2.3	456.0	55.2	2.7	203.0	1.4
STD	0.06	4.94	4.96	0.49	7.28	0.04
DM$_{Hydrolysate}$	3.7	480.0	56.0	< 2.0	129.5	2.3
STD	0.40	0.00	2.00	0.00	13.20	0.25

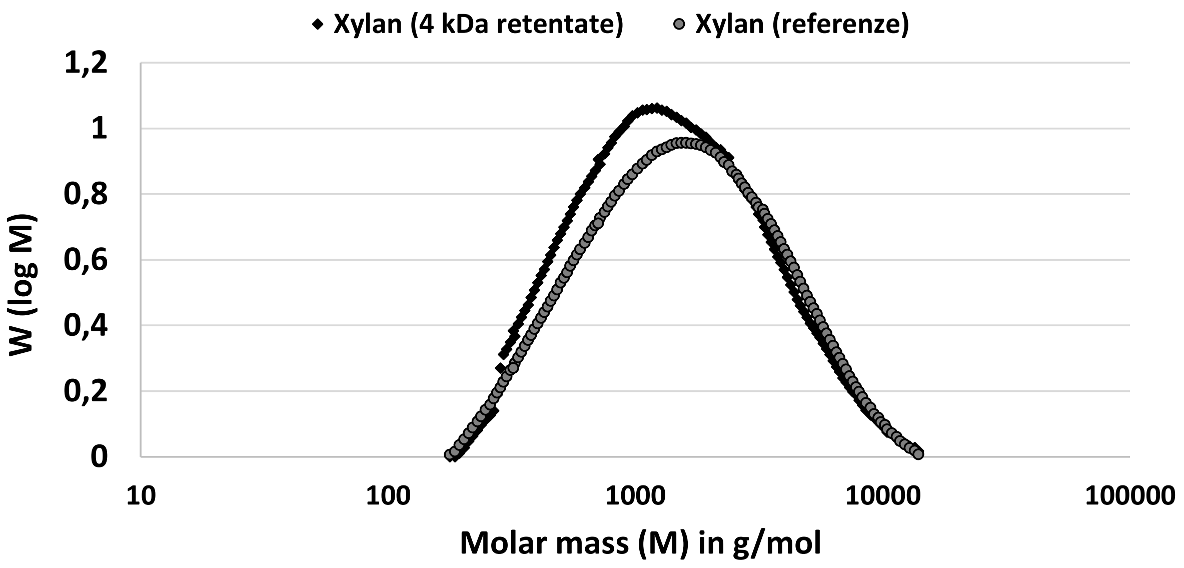

Figure S1: Characterization of oligomers and polymers by size exclusion chromatography (SEC). Distribution of the molecular masses (W (log M) - weighted concentration as a function of the logarithmically scaled molar mass). The 4 kDa retentate sample (filled diamond) was prepared by means of ultrafiltration (4 kDa) and subsequent distillation; the reference sample was prepared only by means of distillation on a laboratory scale. The measurement was carried out at the Institute of Organic Chemistry and Macromolecular Chemistry at the University of Jena. For the measurement (relative to pullulan) the columns NOVEMA 3000 & 300, dimethyl sulfoxide (+ 0.5 % lithium bromide) as eluent and a RI detector were used.

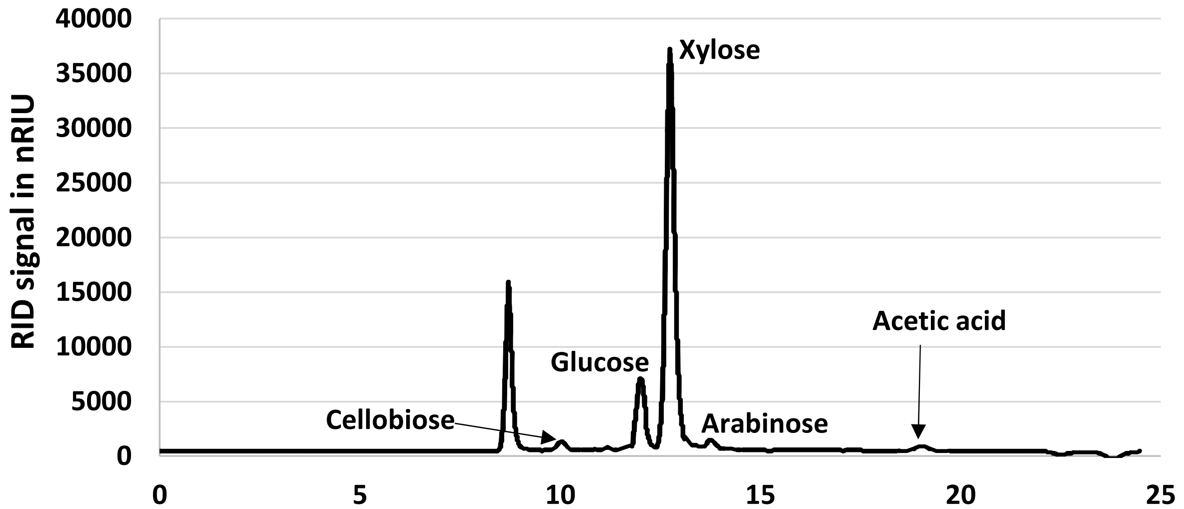

Figure S2: Exemplary xylan chromatogram. Characterization and quantification of xylan in the 4 kDa retentate using HPLC (Agilent Infinity II) and a Hi Plex H+ column.

Table S3: Supplementary information on the filtration of the hydrolysate with a 1 kDa membrane (Pall Omega OM001076). Permeability of the fresh membrane for deionized water (DIW) compared to the fouled membrane.

	Unit	Value	STD
Permeability [a]	l/(m^2 h bar)		
Fresh (DIW)		11.2	0.4

Sample		0.13	0.01
Fouled		1.3	0.1
Backwashed		3.6	0.2
Non-monomeric xylose content	%		
Feed		100.0	--
Retentate		189.6	3.44
Permeate		12.3	3.85
Non-monomeric xylose rejection	%	91.7	--
Non-monomeric xylose filtration yield	%	94.8	--

[a] The membrane with 1 kDa molecular weight cut-off (Pall omega OM001076) has an active layer of polyether sulfone and is therefore presumably less hydrophilic than the other membranes tested, which has an effect on permeability.

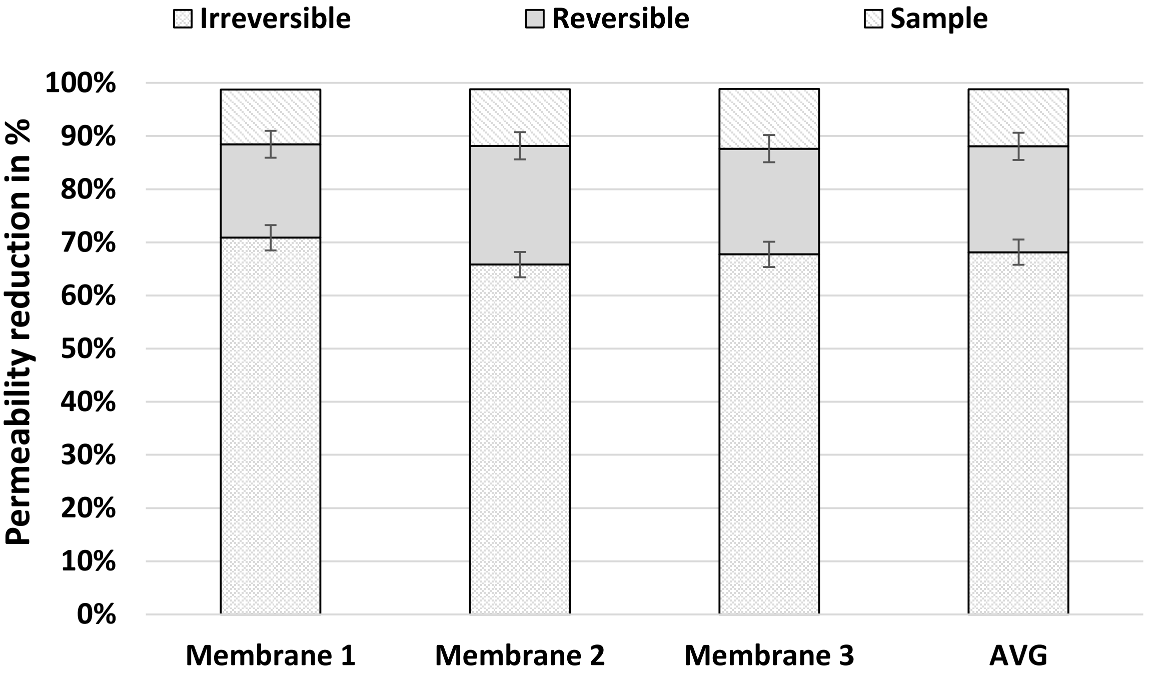

Figure S3: Loss of permeability of the 1 kDa membrane due to fouling effects. Filtration of the hydrolysate sample in comparison to filtration of deionized water with the fresh membrane.

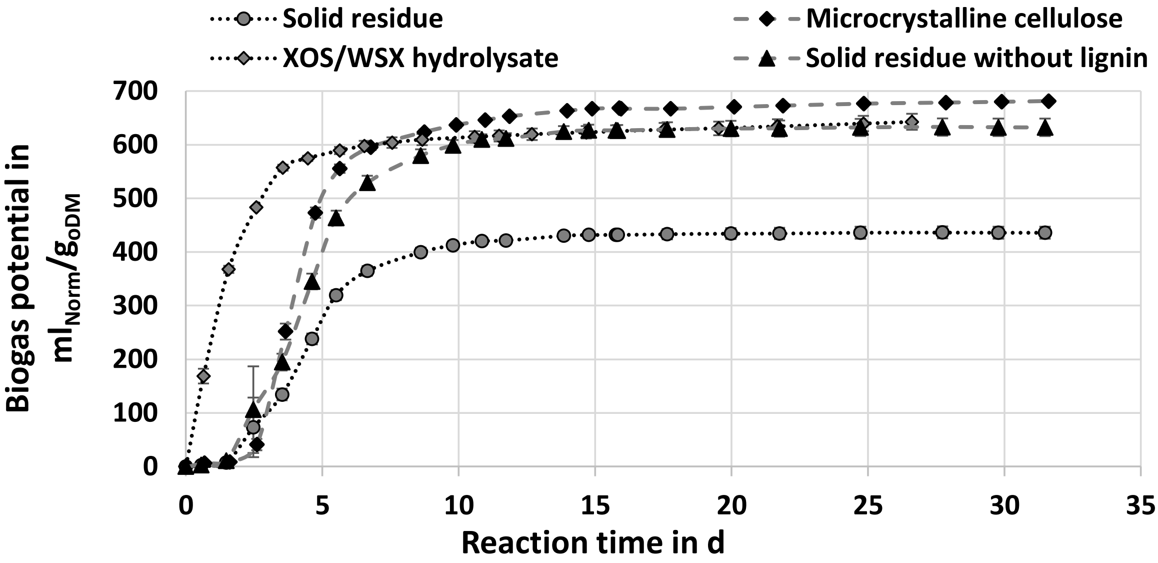

Figure S4: Biogas potential of the hydrolysate and the solid residue. Results and respective standard deviations (error bars) for the solid residue (mainly Cellulose and Lignin) and the hydrolysate in comparison with microcrystalline cellulose. Empty diamonds show the course of the experimentally produced hemicellulose hydrolysate with xylooligosaccharides (XOS) and water-soluble xylan (WSX). Triangles show the calculated course for the solid residue without the lignin fraction; triplicate determinations according to VDI, 2016 (VDI 4630 – Fermentantion of organic materials: Characterisation of the substrate, sampling, collection of material data, fermentation tests). Inoculum: 400 g of digested sludge from a municipal wastewater treatment plant Köhbrandhöft (Hamburg); fermentation in 500 ml bottles at 37 °C; ratio of organic components of the substrate to the organic components of the inoculum was adjusted to 0.3. The analyses were carried out at the Technical University of Hamburg as part of the 031B0660F project.
